# One‐Flask Synthesis of Cyclic Diguanosine Monophosphate (c‐di‐GMP)

**DOI:** 10.1002/cpz1.740

**Published:** 2023-04-11

**Authors:** Barbara L. Gaffney, Roger A. Jones

**Affiliations:** ^1^ Department of Chemistry and Chemical Biology Rutgers University Piscataway New Jersey

**Keywords:** bacterial signaling, c‐di‐GMP, chemical synthesis

## Abstract

The bacterial signaling molecule cyclic diguanosine monophosphate (c‐di‐GMP) plays a key role in controlling biofilm formation and pathogenic virulence, among many other functions. It has widespread consequences for human health, and current research is actively exploring its molecular mechanisms. The convenient one‐flask, gram‐scale synthesis of c‐di‐GMP described here has facilitated these efforts and has been applied to a variety of analogs. © 2023 The Authors. Current Protocols published by Wiley Periodicals LLC.

Basic Protocol 1

A number of routes for the synthesis of c‐di‐GMP have been reported over the years, starting with van Boom's original phosphate triester approach (Ross et al., 1990). Since then, various combinations of amidite and H‐phosphonate couplings have been employed (Amiot et al., 2006; Hayakawa et al., 2003; Hyodo & Hayakawa, 2004; Hyodo et al., 2006; Kiburu et al., 2008; Yan & Aguilar, 2007; Zhang et al., 2004). Until now, these methods have given only small amounts of products. In contrast, the method reported here (Gaffney et al., 2010) yields over 1 g of product and can readily be scaled up further. No chromatographic steps are required, and the intermediate *tert‐*butylammonium salt and the final product **5** are purified by simple crystallizations.

*NOTE*: Reagents and solvents are available from Sigma‐Aldrich, and the apparatus from Fisher.

### Materials

3′‐*O*‐[(Diisopropylamino)(2‐cyanoethoxy)phosphino]‐5′‐*O*‐(4,4′‐dimethoxytrityl)‐2′‐*O*‐*tert*‐butyldimethylsilyl‐*N* ^2^‐isobutyrylguanosine (**1**; Fig. 1)
Dry acetonitrile (CH_3_CN)
3‐Å molecular sieves
Pyridinium trifluoroacetate
*tert*‐Butyl amine (*tert*‐BuNH)
Dichloroacetic acid (DCA)
Pyridine
Ethyl acetate (EtOAc)
Dry nitrogen *or* argon
5.5 M *tert‐*butyl hydroperoxide in decane
Sodium bisulfite (NaHSO_3_)
Sodium bicarbonate (NaHCO_3_)
Dichloromethane (CH_2_Cl_2_)
95% 5,5‐dimethyl‐2‐oxo‐2‐chloro‐1,3,2‐dioxaphosphinane (DMOCP)
Iodine (I_2_)
Diethyl ether (Et_2_O)
Methanol (CH_3_OH)
Potassium hydroxide (KOH)
33% (w/w) methylamine (CH_3_NH_2_) in anhydrous ethanol
Triethylamine (TEA; Et_3_N)
Triethylamine trihydrogen fluoride (Et_3_N·3HF)
Calcium gluconate gel (Lab Safety Supply)
10% (w/w) aqueous potassium carbonate (K_2_CO_3_)
HPLC‐grade acetone

**Figure 1:**
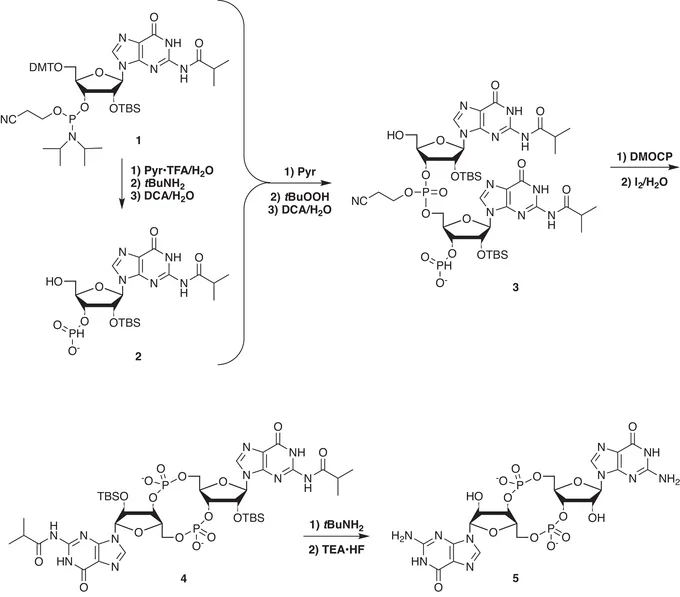
Synthesis of c‐di‐GMP starting from the commercially available guanosine amidite **1**.

100‐ml pear‐shaped flask
Rotary evaporator
Vacuum pump
Septa
100‐ml, 250‐ml, 500‐ml, and 2‐L round‐bottom flasks
Stir bars
Magnetic stir plates
16‐G double‐tipped needle
Disposable syringes and needles
Ice bath
2‐L Erlenmeyer flasks
2‐L separatory funnels
Spatula
Filter paper
Sintered glass funnels
Desiccator
50°C oil bath

Additional reagents and equipment for analytical high‐performance liquid chromatography (HPLC; Sinha & Jung, 2015)

#### Dry compound 1

1 Place 6.31 g (6.5 mmol) of **1** into a 100‐ml pear‐shaped flask. Dry the amidite twice by evaporation of 40 ml CH_3_CN on a rotary evaporator, the last time leaving ∼20 ml. Add ten 3‐Å molecular sieves, and a stopper with a septum. Set aside until needed.

#### Perform hydrolysis, β‐elimination, and detritylation of second portion of 1 to give 2

2 Place another portion of **1** (4.85 g, 5.0 mmol) into a 500‐ml round‐bottom flask with a stir bar. Add 1.16 g of pyridinium trifluoroacetate (6.0 mmol, 1.2 equiv) and rinse down the sides of the flask with 25 ml CH_3_CN. Add 0.18 ml H_2_O (10 mmol, 2 equiv) and stir for 1 min. Add 25 ml *tert‐*BuNH_2_ and let the solution stir for 10 min. Place the flask on a rotary evaporator and concentrate to a foam. Evaporate twice with 50 ml CH_3_CN, each time to give a foam.
  Tert‐BuNH_2_ has a boiling point of 46°C, so it is readily removed.
3 Add 60 ml CH_2_Cl_2_ and mix until the oil is dissolved. Add 0.90 ml H_2_O (50 mmol, 10 equiv) and then 60 ml of 6% dichloroacetic acid (DCA; 44 mmol, 8.8 equiv) in CH_2_Cl_2_.
  CAUTION: Wear gloves, as dichloroacetic acid is very caustic and can remove layers of skin. Upon addition of the acid to the reaction mixture, the solution should turn a deep orange.
  Prepare a total of 140 ml of 6% dichloroacetic acid by adding 8.4 ml dichloroacetic acid to 132 ml CH_2_Cl_2_, because more will be used in step 8. This solution must be used the same day it is prepared.
4 After 10 min, quench with 7.0 ml pyridine (87 mmol, 2 equiv relative to DCA). Concentrate the solution to ∼20 ml and then evaporate three times with 40‐ml portions of dry CH_3_CN, the last time leaving about 12 ml. Check by HPLC (HPLC conditions are described in the Critical Parameters section; retention times are given in Table 1) before the last concentration to be sure detritylation is complete. (If not, concentrate, evaporate several times with ethyl acetate to remove pyridine, and repeat step 3 with less acid, depending on how much tritylated material was left.) Stopper the flask with a septum.
  Upon addition of pyridine, the orange color should immediately disappear.

**Table 1:** HPLC Retention Times for S.1‐S.7

Reverse‐phase HPLC retention time (min)	
**S.1**	11.7^*a*^
**S.2**	5.4^*a*^
**S.3**	9.2/9.5^*a*^
**S.4**	7.2^*a*^
**S.5**	7.3/7.6^*a*^
**S.6**	6.2^*a*^, 9.7^*b*^
**S.7**	3.2^*b*^

^*a*^ Gradient of 2% to 100% CH_3_CN and 0.1 M TEAA.

^*b*^ Gradient of 2% to 40% CH_3_CN and 0.1 M TEAA.

#### Perform linear coupling of 1 with 2 and detritylation to give 3

5 Transfer the dried **1** to **2** through a 16‐G double‐tipped needle by inserting one end of the needle through the septum of the flask containing **1** all the way to the bottom, and the other end through the septum of the flask containing **2** but above the solution. Place a vent needle in the flask with **2**. Place a needle‐tipped line from a source of dry nitrogen or argon into the flask with **1**, thereby causing the solution of **2** to flow into the flask with **1**.
  Examples are shown in Sigma‐Aldrich technical bulletins (http://www.sigmaaldrich.com/chemistry/chemical‐synthesis/learning‐center/technical‐bulletins.html): AL‐134 (Handling Air‐Sensitive Reagents) and AL‐164 (Handling Pyrophoric Reagents).
  It is best to have practiced this transfer ahead of time using only CH_3_CN. The nitrogen pressure should be such that the solution of **1** is transferred in <1 min.
6 Immediately inject a 1‐ml rinse of dry CH_3_CN into the flask that had contained **1** (from step 1), rotate the flask quickly to rinse the sides of the flask, and transfer this rinse through the double‐tipped needle into the flask with **2**. Repeat this rinse one more time with 1 ml fresh CH_3_CN. Allow the mixture of **1** and **2** to stir for 2 min, and then take a sample for HPLC analysis (HPLC conditions are described in the Critical Parameters section; retention times are given in Table 1).
7 As soon as the HPLC sample is removed, add 2.73 ml anhydrous 5.5 M *tert‐*butyl hydroperoxide in decane (15 mmol, 3 equiv). Let the solution stir for 30 min and then take an HPLC sample. Once the HPLC sample is removed, place the flask in an ice bath and add 1.25 g NaHSO_3_ dissolved in 2.5 ml H_2_O. After 5 min of stirring, concentrate to an oil.
  The anhydrous tert‐butyl hydroperoxide should be kept in a refrigerator but removed an hour before use. The NaHSO_3_ solution takes some time to dissolve, so prepare it 15 min ahead of time, but not earlier.
8 Add 80 ml CH_2_Cl_2_ to the flask and mix until the oil is dissolved. Add 0.90 ml H_2_O (50 mmol, 10 equiv) and then 80 ml of 6% dichloroacetic acid (58 mmol, 11.6 equiv) in CH_2_Cl_2_.
  Use the rest of the 6% dichloroacetic acid prepared in step 3.
9 After 10 min, quench the reaction with 15 ml pyridine. Take an HPLC sample, add 35 ml more pyridine, and concentrate to ∼20 ml. Add 150 ml pyridine and concentrate to 100 ml. Stopper the flask with a septum.
  The orange color for this detritylation is often less intense than in step 3.

#### Perform cyclization of 3 to give 4

10 Add 3.40 g of 95% 5,5‐dimethyl‐2‐oxo‐2‐chloro‐1,3,2‐dioxaphosphinane (DMOCP; 17.5 mmol, 3.5 equiv) and stir for 10 min.
  DMOCP is also called 2‐chloro‐5,5‐dimethyl‐1,3,2‐dioxaphosphorinane 2‐oxide.
11 Add 3.2 ml H_2_O (175 mmol, 10 equiv relative to DMOCP). Immediately add 1.65 g I_2_ (6.5 mmol, 1.3 equiv) and stir for 5 min. Take an HPLC sample, immediately pour the mixture into a 2‐L Erlenmeyer flask containing 700 ml H_2_O and 1.0 g NaHSO_3_, and stir 5 min.
12 Slowly add 20 g NaHCO_3_ (238 mmol) and stir for 5 min.
  The mixture will foam.
13 Add 800 ml of 1:1 (v/v) EtOAc/Et_2_O and stir for 5 min. Pour the mixture into a 2‐L separatory funnel and shake thoroughly. Drain the aqueous layer and pour the organic layer into a 2‐L round‐bottom flask. Extract the aqueous layer again with an additional 200 ml of 1:1 EtOAc/Et_2_O. Drain the aqueous layer and pour the second organic layer into the round‐bottom flask. Concentrate to remove most of the ether, and check by HPLC.
  At this point, the flask can be placed in a freezer overnight and the work continued the next day.

#### Perform deprotection of 4 to give 5

14 Continue concentrating the solution of **4** to less than 50 ml. Transfer the solution into a 250‐ml round‐bottom flask. Add 20‐ml portions of ethyl acetate and evaporate three times to help remove excess pyridine.
15 Add a stir bar and 25 ml CH_3_CN. Stir to mix, add 25 ml of *tert‐*BuNH_2_, and stir for 10 min. Concentrate the solution to dryness. Add 25 ml CH_3_CN, scrape the sides of the flask with a spatula, and concentrate to dryness. Repeat two more times. Dissolve the solid in 25 ml CH_3_OH and check by HPLC. Filter (using a filter paper) the solution into a 100‐ml round‐bottom flask, removing the stir bar. Concentrate the solution to a foam.
  As the β‐elimination takes place, the product forms as a white solid. Thorough mixing of this solid upon each addition of CH_3_CN will help to remove the tert‐BuNH_2_.
16 Add 25 ml CH_2_Cl_2_ to the foam and immediately swirl vigorously until the solid dissolves. Stopper the flask with a septum, let it sit until crystal deposition has slowed, and then refrigerate overnight.
  Crystallization should begin anywhere from 1 min to 1 hr, and should be nearly complete after 30 min.
17 Collect the white crystals of the *tert‐*butyl ammonium salt in a preweighed sintered glass funnel and wash them three times with no more than 2 ml CH_2_Cl_2_ each time, stirring thoroughly. Cover the funnel with filter paper and dry it in a desiccator over KOH overnight. Weigh it and check a sample by HPLC.
  The melting point should be 191°‐193°C dec. The NMR should be as follows: ^1^H NMR (DMSO‐d_6_) 55°C δ 8.24 (s, 2H), 5.88 (d, J = 3 Hz, 2H), 4.93 (br, 2H), 4.38 (br, 2H), 4.14 (br, 2H), 4.10‐4.03 (m, 2H), 3.86‐3.79 (m, 2H), 2.83 (sep, J = 7 Hz, 2H), 1.22 (s, 22H), 1.06 (d, J = 7 Hz, 6H), 0.91 (d, J = 7 Hz, 6H), 0.84 (s, 18H), 0.14 (s, 6H), 0.07 (s, 6H); ^13^C NMR (DMSO) 55°C δ (all resonances are singlets) 182.0, 156.4, 149.9, 149.5, 139.5, 122.3, 91.0, 82.4, 77.6, 72.7, 63.3, 52.3, 28.7, 27.4, 20.4, 20.1, 19.4, –2.9, –3.8; ^31^P NMR (DMSO‐d_6_) 55°C δ –1.87.
18 Transfer the above into a 250‐ml round‐bottom flask. For each 1.00 mmol (1.20 g), add 100 ml CH_3_NH_2_ (800 equiv) in anhydrous ethanol (33% by weight). Add a stir bar, cap with a septum, and stir for 90 min. Check completion of the reaction by HPLC. Concentrate to an oil. Add 4 ml pyridine and 2 ml Et_3_N, swirl to mix, and concentrate to an oil. Repeat this procedure two more times.
  Because tert‐BuNH_2_ (bp 46°C) is more volatile than Et_3_N (bp 89°C), the tert‐BuNH_2_ is removed by this process, thereby converting the salt form from tert‐BuNH_3_^+^ to Et_3_NH^+^.
19 Add 4 ml pyridine to the flask, cap with a septum, and insert a vent needle. Place the flask in an oil bath at 50°C in a hood and stir. For every 1.00 mmol (1.20 g), draw up 14 ml Et_3_N and 8.3 ml Et_3_N·3HF (150 equiv F^–^) into separate syringes and insert them both through the septum of the flask. Add the two reagents simultaneously over ∼1 min. Stir the mixture for 1 hr at 50°C, occasionally rotating the flask at an angle to dissolve solids on the sides.
  CAUTION: HF is extremely dangerous and can cause severe burns. Wear heavy gloves for this procedure. Do not breathe vapors from the flask or allow any Et_3_N·3HF to contact your skin. When done, rinse all needles, syringes, septa, etc. with aq. K_2_CO_3_ before discarding. Keep a supply of calcium gluconate gel (available from Lab Safety Supply) at hand for emergency treatment, should any Et_3_N·3HF contact your skin.
20 Remove the flask from the oil bath and start it stirring on a different stir plate. Remove the septum, and for every 1.00 mmol, add 112 ml HPLC‐grade acetone in a slow, steady stream over 1 min. Continue to stir the mixture as the product crystallizes. After 10 min, collect the crystals by filtration in a sintered glass funnel and wash them five times using 5‐ml portions of acetone. Stir thoroughly between each wash. If the product is sticky, use a spatula to repeatedly smear it against the sides of the funnel, thereby removing trapped impurities. Cover the funnel with filter paper and dry it in a desiccator over KOH overnight. Weigh it and check a sample by HPLC. Analyze **5** by ^1^H and ^31^P NMR.
  The melting point should be 193°‐196°C dec. The NMR should be as follows: ^1^H NMR (D2O) 55°C δ 8.03 (s, 2H), 5.81 (s, 2H), 5.07 (br, 2H), 4.80 (br, 2H), 4.07‐3.96 (m, 2H), 3.13 (q, J = 7 Hz, 12H), 1.22 (t, J = 7 Hz, 18H); ^13^C NMR (D2O) 55°C δ (all resonances are singlets)159.3 (br), 156.4, 152.1 (br), 139.1, 117.5 (br), 92.8, 83.0, 75.8, 73.0, 65.0, 49.4, 10.8; ^31^P NMR (D2O) 55°C δ –0.20.
  Take great care when handling the acetone filtrate and washes because they contain excess Et_3_N·3HF.

## COMMENTARY

### Background Information

The bacterial signaling molecule cyclic diguanosine monophosphate (c‐di‐GMP) plays key roles in controlling biofilm formation, organelle formation for motility, cell‐cycle differentiation, and pathogenic virulence, among many other functions (Cotter & Stibitz, 2007; Hengge, 2009; Romling & Amikam, 2006; Schirmer & Jenal, 2009; Tamayo et al., 2007). It acts by relaying extracellular signals to internal receptors, including PilZ proteins (Ko et al., 2010), as well as two different classes of riboswitches (Kulshina et al., 2009; Lee et al., 2010; Smith et al., 2009, 2011; Sudarsan et al., 2008). It has widespread consequences for human health, and current research is actively exploring its molecular mechanisms. The convenient one‐flask, gram‐scale synthesis of c‐di‐GMP described here and shown in Figure 1 has facilitated these efforts and has been applied to a variety of analogs (Bartsch et al., 2022; Chen, 2023; Chen et al., 2023; Gao, Ascano, Wu, et al., 2013; Gao, Ascano, Zillinger, et al., 2013; Meehan et al., 2016; Sun et al., 2023; Yan, 2021; Zhang et al., 2013, 2023).

### Critical Parameters

The detritylations in steps 3 and 8 are potentially reversible upon quenching and again upon concentration, in spite of the water added to prevent it. The concentrated oils of detritylated **2** and **3** should be used immediately, and not stored.

Conditions for the initial amidite coupling between **1** and **2** should be as dry as possible.

HPLC is useful for following the course of the synthesis. We used a Waters Atlantis C18 column, 4.6 mm × 50 mm, 3 μm, with gradients of 2% to 100% CH_3_CN and 0.1 M triethylamine acetate (TEAA; pH 6.8) for steps 1 to 17, and 2% to 40% at the end of step 18. However, many steps are time dependent, and the procedure must be continued immediately after taking samples, without waiting to see the results. Note that compounds with a cyanoethyl group may elute as two peaks because there are two diastereomers.

The linear dimer is best cyclized and oxidized as soon as it is made. Therefore, steps 1 to 13 should be performed on the same day.

Because of the danger of using Et_3_N·3HF, HPLC should not be performed to monitor progress of the final desilylation. Once the solids are in solution (there may be two layers), the reaction should go to completion in 1 hr.

### Anticipated Results

The yield for crystallization of the intermediate *tert*‐butylammonium salt should be ∼35% from **1**, and the yield for crystallization of the final product, **5**, should be ∼30% from **1**. Both are white powders that are homogeneous by HPLC‐mass spectrometry (HPLC‐MS).

### Time Considerations

The entire sequence of reactions can be done in 4 days, with final NMR analysis taking longer. Steps 1 to 13 should be performed all in 1 day (8 to 10 hr) or the yield will suffer. Steps 14 to 16 take a second day (2 to 3 hr), and step 17 takes only a few minutes of a third day. Steps 18 to 20 take a fourth day (6 to 8 hr).

### Author Contributions

**Barbara L. Gaffney**: Investigation, writing—original draft, writing—review and editing; **Roger A. Jones**: Conceptualization, funding acquisition, project administration, writing—original draft, writing—review and editing.

### Conflict of Interest

The authors declare no conflict of interest.

## Data Availability

No new data were created or analyzed during the writing of this protocol.
